# Relationship Between Executive Function and Motor Skills in Early Childhood: A Longitudinal Study

**DOI:** 10.3390/bs16081291

**Published:** 2026-07-28

**Authors:** Bo Lyu, Zhixuan Liu, Xiaozi Gao

**Affiliations:** 1 Faculty of Education, Northeast Normal University, Changchun 130024, China; lzxanny@126.com; 2 Department of Early Childhood Education, The Education University of Hong Kong, Hongkong, China; gaox@eduhk.hk

**Keywords:** executive function, preschoolers, gross motor, fine motor, RI-CLPM

## Abstract

This longitudinal study investigates the dynamic interplay between Executive Function (EF), gross motor (GM), and fine motor (FM) skills in early childhood, specifically examining their mutual influences over a three-year period. The study involved 2164 children (1113 boys, 1051 girls) at Time Point 1 (T1) when they were 3 years old, with follow-up assessments at T2 (age 4) and T3 (age 5). Data were collected through parent-reported measures and analyzed using Random Intercepts Cross-Lagged Panel Models (RI-CLPM) to examine the temporal relationships between these variables. Our findings reveal significant autoregressive effects for EF and FM skills from T1 to T3, indicating that early performance in these areas predicts subsequent performance. Notably, FM skills demonstrated a significant cross-lagged effect on both EF and GM skills across all three assessment waves. The study underscores the potential benefits of combined EF and motor skills training for enhancing developmental outcomes in preschool children.

## 1. Introduction

Early childhood represents a critical window for developmental cascades. During this period, children experience rapid maturation in higher-order cognitive processes—collectively known as executive functions (EF)—which enable goal-directed behaviors, problem-solving, and reasoning (Diamond, 2013; Garon et al., 2008; Munakata et al., 2012). Concurrently, the neurocognitive and physiological systems supporting these abilities undergo substantial refinement (Best & Miller, 2010; Diamond, 2000). Specifically, at the neural circuit level, the prefrontal-striatal and prefrontal-cerebellar pathways undergo accelerated myelination and functional specialization. At the neuromodulator level, the dopamine system synchronously regulates motor control and EF. At the developmental timing level, protracted maturation of the prefrontal cortex and neocerebellum, combined with heightened plasticity, creates a critical window for synergistic motor-EF development. This systemic maturation not only facilitates the progression of EFs but also drives the continuous development of motor skills. A growing body of literature indicates a strong concordance between motor and cognitive trajectories (Chichinina et al., 2025; Diamond, 2000; Houwen et al., 2017; Michel et al., 2016; Roebers et al., 2014), suggesting intertwined neural foundations and developmental mechanisms. However, the causal directionality between the two remains widely debated: no consensus has been reached in the literature regarding whether EF and motor skills exhibit bidirectional reciprocal prediction or asymmetric unidirectional influence. Clarifying this issue can not only deepen our understanding of the principles of child development, but also provide a scientific basis for early education and intervention practices.

Motor skills are critical for early interaction with the physical and social environment (Hofsten, 2009; Wilson, 2002). They are typically categorized into Gross Motor skills (GM), involving large muscle groups for whole-body movement and posture (Bardid et al., 2016), and Fine Motor skills (FM), involving precise, goal-directed movements of small muscle groups (Piek et al., 2008). Existing research has shown a high degree of concordance between the developmental trajectories of motor and cognitive functions (Chichinina et al., 2025; Michel et al., 2016). The two domains share a core neural circuit centered on the dorsolateral prefrontal cortex, the cerebellum, and their connecting fibers. However, the strength of the neural associations with EF differs between the two types of motor skills: compared with GM tasks, FM tasks exhibit stronger functional connectivity with the prefrontal cortex and impose higher cognitive demands on visuomotor integration and action regulation (Stoodley, 2011).

Although neuroscientific evidence supports an intrinsic motor-EF association, theoretical frameworks diverge sharply on causal direction of this relationship—a tension that directly motivates the present study. The concept of motor-cognition associations is historically grounded in Piaget’s theory, which posits that the development of sensorimotor and motor skills serves as a foundation for cognitive advancement (Piaget, 1952). Embodied Cognition Theory further argues that cognitive abilities are rooted in physical interactions with the environment (Foglia & Wilson, 2013). Thus, motor development acts as a catalyst for cognitive progress, facilitating learning through “perception-action” cycles (Hofsten, 2007). These theories support a unidirectional motor-to EF pathway. In contrast, Dynamic Systems Theory posits that motor skills and EF exert continuous, bidirectional influences within an integrated system (Thelen, 2005). Although two core theoretical positions have emerged, empirical research has yet to provide sufficiently rigorous longitudinal evidence to test them. As a result, no consensus has been reached on the causal direction between motor skills and EF.

The preschool period is a key developmental window: EF and motor skills undergo rapid, synchronized development. The prefrontal cortex (the core brain region for EF) enters a period of active gray matter expansion and synaptic pruning, while the neural connections between the motor cortex and the cerebellum are also undergoing rapid refinement. The synergistic developmental effects of these processes are most pronounced during this period (Diamond, 2000; Huttenlocher, 1979), making it the optimal time to capture their dynamic interplay. Methodologically, EF remains relatively undifferentiated during the preschool years. Although the tripartite model of EF is widely accepted, evidence in preschool children indicates that prefrontal neural circuits are not yet fully differentiated at this stage. A systematic review shows that cognitive flexibility failed to emerge as a separate factor in preschoolers (Karr et al., 2018). Near-infrared spectroscopy (NIRS) studies have also demonstrated that the prefrontal activation patterns of 4- to 6-year-old children are highly similar during working memory and inhibitory control tasks (Tsujimoto, 2008). Longitudinal latent variable analyses have further confirmed the high cross-time consistency of a unidimensional EF structure in this age group (Willoughby et al., 2012). This undifferentiated nature allows researchers to more clearly examine the dynamic interplay between motor skills and EF, without the confounding influence of differentiated EF components.

Considerable evidence examines EF associations with both motor skills. The relationship between FM and EF is a core topic in developmental science (C. Chen et al., 2026; Karle et al., 2026). Cross-sectional studies have found a stable positive association between the two in children aged 3–6 years (Ashkenazi & Adi, 2023; Becker et al., 2014; Gandotra et al., 2022). Longitudinal studies, however, further reveal an asymmetric pattern: early FM skills significantly predict later EF. This pathway has been validated in children aged 5–7 years (Brock et al., 2018; Oberer et al., 2018) and in Arabic-speaking children around age 6 (Khoury-Metanis & Khateb, 2024). In contrast, the reverse predictive relationship has not been observed in longitudinal studies of children aged 3–5 years (MacDonald et al., 2016) or 4–6 years (Michel et al., 2020).

Similarly, GM skills are associated with EF, though findings vary by gender and BMI, with effects reported in children aged 3–5 years (Fernandes et al., 2022) and 9–11 years (Aadland et al., 2017; Liu et al., 2022). In terms of longitudinal research, Piek et al. (2008) tracked children from 4 months to 11 years, and M. Wu et al. (2017) tracked children from 1 to 3 years; both found that early GM predicts later cognitive outcomes. However, latent variable models in 3–6 year-olds suggest equally strong bidirectional effects (Zysset et al., 2020). This discrepancy highlights the necessity of rigorous methodological approaches to clarify these relationships.

Studies examining FM, GM and EF in the same framework also yield inconsistent findings (Gandotra et al., 2023; Karle et al., 2026; Leisman et al., 2026; Oberer et al., 2017; Sung et al., 2024). Among preschool-aged children (3–6 years), some studies report a stronger FM–EF association (Sung et al., 2024), whereas others find a stronger overall GM–EF association in children aged 5–7 years (Oberer et al., 2017). At school age (6–12 years), the available evidence more consistently points to a stronger FM–EF association (Policastro et al., 2018). This age-related inconsistency suggests that the patterns of association among FM, GM, and EF may undergo dynamic changes across developmental stages. Although prior research has explored this issue, two key limitations remain.

First, no consensus has been reached on the dynamic associations among FM, GM and EF (Gandotra et al., 2023; Karle et al., 2026; Leisman et al., 2026; Oberer et al., 2017; Sung et al., 2024). Most existing studies that integrate EF, GM, and FM rely on cross-sectional designs, which can only capture concurrent covariation at a single time point. Such designs cannot determine the temporal precedence or causal direction among these three domains (Houwen et al., 2017), nor can they disentangle whether divergent findings across studies reflect genuine age-related differences or merely sample characteristics. Therefore, longitudinal tracking designs are needed to clarify the longitudinal predictive relationships and directional pathways among FM, GM, and EF, addressing the methodological shortcomings of prior cross-sectional research.

Second, cultural representativeness remains inadequate. Most existing developmental models have been constructed based on Western samples, yet cultural background significantly moderates both the developmental levels of these skills and the strength of their interrelationships (Sung et al., 2024; Xu et al., 2023). Research has shown that Chinese preschool children tend to outperform their Western peers on EF tasks such as inhibitory control (Lan et al., 2011; Sabbagh et al., 2006) and in FM domains such as manual dexterity (Chow et al., 2001; Chui et al., 2007), whereas Western children demonstrate advantages in object-control GM skills (Ma et al., 2022). Against the backdrop of markedly different educational practices and motor experiences in the Chinese cultural context, verifying the universality of motor–cognition co-development theories and supplementing the literature with large-sample longitudinal evidence from non-Western cultures represent an urgently needed direction for future research in this field.

**Figure 1:**
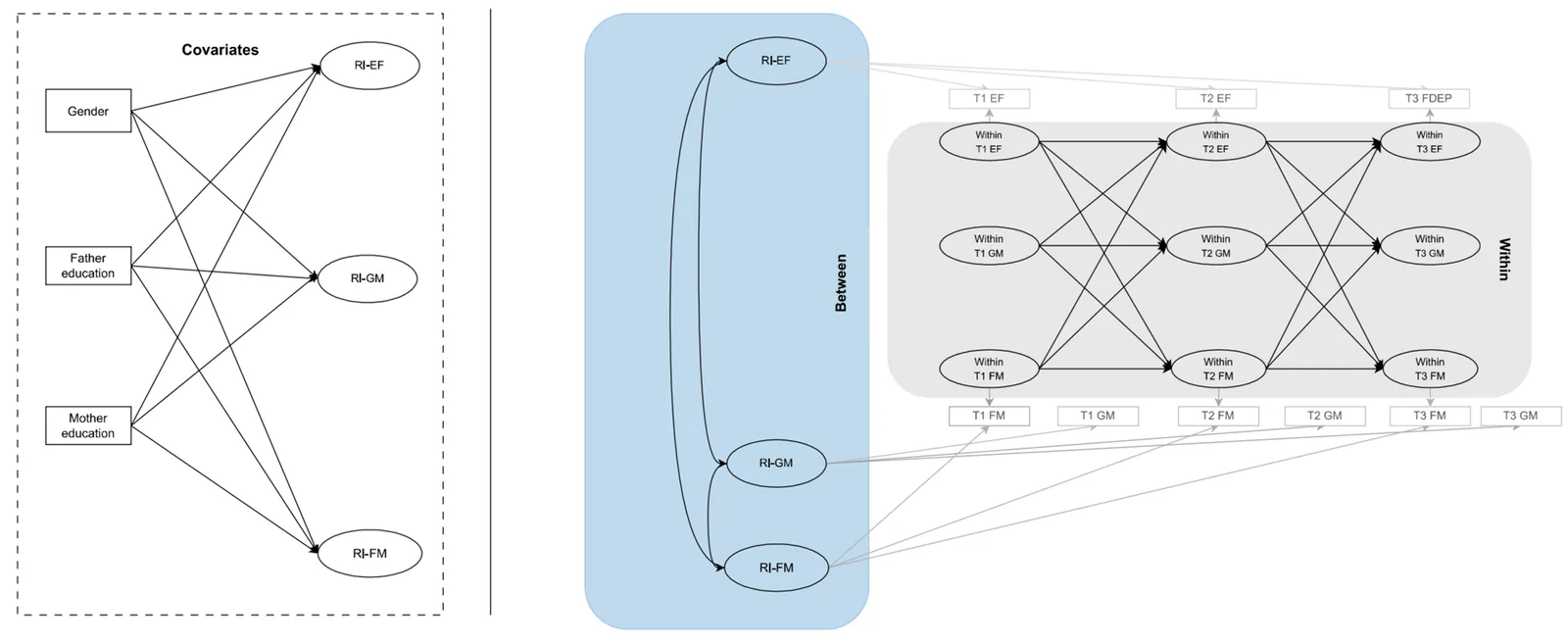
Hypothetical Model. Note: EF = executive function; GM = gross motor skills; FM = fine motor skills; RI = random intercept; T1, T2, T3 = first, second, and third measurement time points.

On this basis, the present study uses a three-wave longitudinal design with RI-CLPM to separate trait-like between-person covariation from within-person dynamic effects, clarifying the causal direction among EF, GM skills, and FM skills during the preschool period (See Figure 1). It provides empirical evidence from a Chinese cultural sample to test the applicability of embodied cognition theory and dynamic systems theory, further refining the theoretical framework of motor-cognition co-development. At the practical level, the findings can offer an evidence-based pathway for optimizing kindergarten motor curricula and designing early cognitive intervention programs, providing data support for an educational approach that integrates motor and cognitive development during the preschool years. After controlling for child sex and parental education level, the following hypotheses are proposed:

**H1.**  *EF, GM, and FM skills will demonstrate significant autoregressive effects.*

**H2.**  *Within-person changes in preschoolers’ FM skills will significantly predict subsequent within-person changes in EF, and vice versa.*

**H3.**  *Within-person changes in preschoolers’ GM skills will significantly predict subsequent within-person changes in EF, and vice versa.*

**H4.**  *The between-person effects of EF, GM skills, and FM skills will be significantly correlated with each other.*

## 2. Methods

### 2.1. Participants

This study adopted a longitudinal tracking design, with repeated measurements at three time points—when children were 36, 48, and 60 months of age—to examine the dynamic developmental relationships among FM skills, GM skills, and EF. Data were drawn from the “Kids in Taiwan: National Longitudinal Study of Child Development and Care” (KIT) project (Chang, 2019). The sampling frame was the household registration system in Taiwan, encompassing all children born between 1 April 2013 and 31 March 2014. A stratified two-stage probability proportional-to-size (PPS) sampling method was used to ensure the sample’s representativeness across urbanization, population structure, and density in Taiwan.

This study utilized data from three waves: Time 1 (T1, 2016–2017) was collected when children were approximately 36 months old, Time 2 (T2, 2017–2018) at 48 months, and Time 3 (T3, 2018–2019) at 60 months. The baseline (T1) sample consisted of 2164 children (1113 boys and 1051 girls; M = 3.19, SD = 0.39). Follow-up assessments included 2031 children at T2 (1029 boys and 1002 girls), and 1985 children (1018 boys and 967 girls) at T3 (retention rate = 91.7%). At baseline, 49% of fathers and 48% of mothers had a high school education or lower, 32% of fathers and 40% of mothers held a university degree, and 16% of fathers and 11% of mothers had acquired postgraduate qualifications. The vast majority of parents were of Chinese nationality (97.9% of fathers, 95.8% of mothers).

### 2.2. Procedures

Ethical approval for this study was granted by National Taiwan Normal University(Approval No. 201707HS003). Our analysis focused on the parent-report questionnaires. A standardized development procedure was used to reduce parental comprehension bias, and behaviorally anchored, age-graded rating scales were employed to specifically address the subjectivity inherent in rating-based assessments. The validity of parent ratings was further empirically verified through multidimensional reliability and validity tests. To ensure that parents completed the questionnaires within the designated assessment window, thereby reducing recall bias and errors arising from temporal misalignment, questionnaires were administered during scheduled in-home interviews. To ensure longitudinal consistency, the same caregiver was required to provide data at all three time points.

### 2.3. Measures

#### 2.3.1. Child EF

The study assessed children’s EF development using the “Cognitive Development Parental Questionnaire for 2- to 5-Year-Olds” (S. M. Wang et al., 2015). In the present study, EF was operationalized as children’s everyday behavioral regulation abilities, rather than core cognitive-level EF. This parent-report questionnaire has demonstrated strong reliability and validity in Chinese populations. The EF scale comprises 6 items rated on a 4-point scale (1 = not at all, 4 = very proficient), and assesses EF through observable behaviors in daily life scenarios. For example, items include “In public, when you ask your child to lower his/her volume, he/she can immediately lower volume and maintain it for at least a few minutes” and “After your reminding, your child can slow down and make things better.” Higher scores indicate stronger EF abilities. In this study, Cronbach’s α coefficients were 0.88 at T1, 0.88 at T2, and 0.88 at T3.

#### 2.3.2. Child Motor Skills

The assessment of children’s motor skill development employed the Developmental Motor Screening Scale for Preschool Children (DMSSPC; Po et al., 2016; C. C. Wang et al., 2015). This standardized instrument contains 46 items measuring both GM and FM skills reported by parents. The GM domain comprises two subdimensions: stability and locomotion (8 items) and body coordination (14 items). The FM domain includes grasp and manipulation (12 items) and visual-motor integration (12 items). Items were rated on a 4-point scale (1 = not at all, 4 = very proficient), assessing abilities such as “Standing while throwing a small ball overhand with one hand”. Parents evaluated their children’s motor skills based on daily performance, with higher scores indicating better motor skills. Reliability analysis demonstrated strong internal consistency across three waves: For the GM scale, Cronbach’s α coefficients were 0.876 at T1, 0.873 at T2, and 0.878 at T3. For the FM scale, the Cronbach’s α coefficients were 0.88 at T1, 0.87 at T2, and 0.87 at T3.

### 2.4. Data Analysis

All analyses were conducted using SPSS 26.0 and Mplus 8.0. First, we assessed common method bias using Harman’s single-factor test (Podsakoff et al., 2003). Then, we conducted independent samples t-tests to compare children with missing data at T2/T3 and those with complete longitudinal data for group differences. We further performed MCAR test (Little & Rubin, 1987). Given that the conventional Little’s MCAR chi-square test is overly sensitive to large sample sizes and datasets with a large number of items, even trivial differences that have no practical impact on parameter estimation can result in a statistically significant test (Enders, 2010). Accordingly, the normed chi-square (χ^2^/df) was adopted as the evaluation criterion. The Expectation-Maximization (EM) algorithm was employed to handle missing values (Ullman & Bentler, 2012).

Second, we computed descriptive statistics, including means, standard deviations, and bivariate correlations for the main variables of interest across all three waves.

Recent statistical advancements emphasize the distinction between between-person (trait) and within-person (state) effects. Conventional Cross-Lagged Panel Models (CLPM) fail to separate these variances, potentially confounding stable individual differences with developmental dynamics (Berry & Willoughby, 2017; Hamaker et al., 2015). As a result, we employed a RI-CLPM to explore the dynamic interplay between EF, GM skills, and FM skills over time, following the procedures outlined by Mulder and Hamaker (2020). The RI-CLPM enhances the CLPM by incorporating random intercepts for each variable, which partitions the variance into two distinct components: within-person and between-person variance (Hamaker et al., 2015). The random intercepts for each construct represent the stable, trait-like component consistent across the three waves. After the separation of these random intercepts, the residual variation represents the within-person fluctuations, thereby allowing for an evaluation of the temporal dynamics and fluctuations around these stable, trait-like components at the individual level. Within-person concurrent associations were all freely estimated (Orth et al., 2021). Child sex and parental education level were included as covariates for all observed variables at T1 as well as for the random intercepts.

Guided by Orth et al. (2021) and in the interest of model parsimony, we conducted a sequence of model comparisons. We tested four nested models: Model 1 established the baseline for comparison by allowing all parameters to be estimated freely, without any constraints. Model 2 applied constraints to the autoregressive paths for each variable across the waves. Model 3 applied constraints to the cross-lagged paths between variables across time points. Model 4 applied constraints to both autoregressive and cross-lagged paths over time.

Nested models were compared using the χ^2^ difference test. However, due to its sensitivity to large sample sizes (Brannick, 1995; Curran et al., 2002), we also considered other commonly used fit indices in evaluating model fit (F. Chen, 2007; Cheung & Rensvold, 2002). A model is generally considered acceptable when the CFI exceeds 0.90 and is close to 0.95, the RMSEA is 0.08 or lower, and the SRMR is 0.05 or lower (Bentler & Bonett, 1980; Hu & Bentler, 1999).

## 3. Results

### 3.1. Preliminary Statistics

First, Harman’s single-factor test (Podsakoff et al., 2003) was used to assess common method bias. The first factor of the three waves of data accounted for 22.17%, 23.93%, and 26.85% of the total variation, respectively, all lower than 40%, indicating that there was minimal risk of common method bias. Next, independent samples *t*-tests revealed no significant differences between the two groups on all key variables (*ps* > 0.05), indicating that missing longitudinal data were not attributable to differences in children’s baseline developmental levels. We further performed MCAR test (Little & Rubin, 1987). The overall normed χ^2^/df was 1.22, which was below the critical cutoff of 2.

### 3.2. Descriptive Statistics and Correlations

The descriptive statistics and bivariate correlations of the mean scores at the three time points are presented in Table 1, where gender was coded as 1 for male and 2 for female. The table demonstrates that the associations among EF, GM, and FM were significant across the three time points, indicating a consistent relationship between these variables throughout the preschool period. The participants included in this study were all typically developing children. Preterm births accounted for only 4.2% of the sample. The majority of deliveries were vaginal births (57.9%). Children’s BMI values were concentrated in the 14–16 kg/m^2^ range, consistent with the normal growth and development of children in this age group.

### 3.3. Random Intercept Cross-Lagged Panel Models (RI-CLPM)

**Figure 2:**
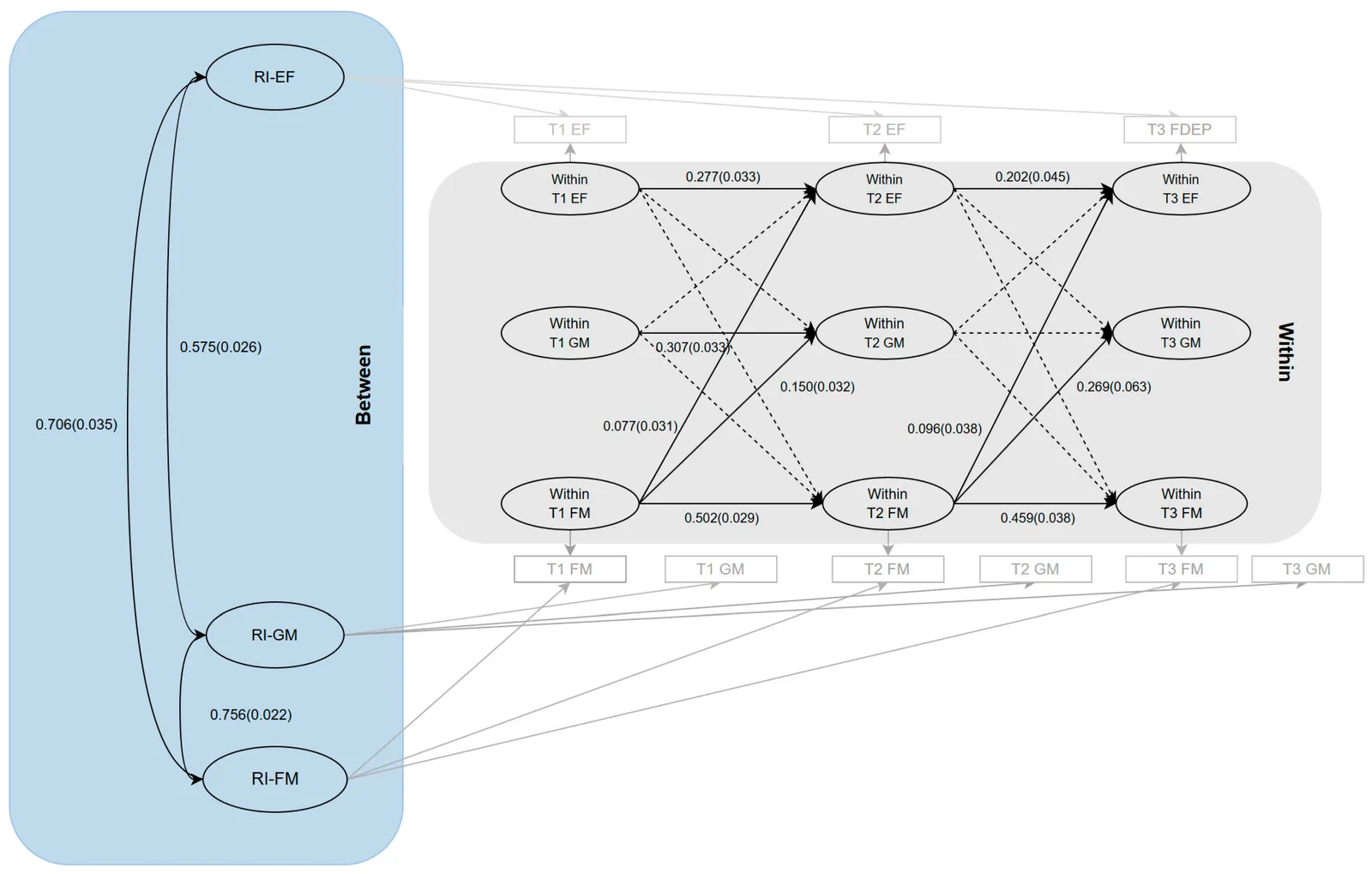
Random Intercept Cross-Lagged Panel Model. Note: EF = executive function; GM = gross motor skills; FM = fine motor skills; RI = random intercept; T1, T2, T3 = first, second, and third measurement time points.

Considering different goodness-of-fit indices, the model building procedure indicated Model 3 (χ_(9)_^2^ = 45.157, RMSEA = 0.041, CFI = 0.997, TLI = 0.989, SRMR = 0.021) as the more parsimonious model (see Table 2). Specifically, the chi-square difference between M3 and M1 was non-significant (Δχ^2^ = 16.376, Δdf = 6, *p* = 0.012). These results indicate that imposing time-invariant constraints on the cross-lagged paths did not significantly worsen model fit while effectively improving model parsimony. At this point, we added the covariates to the final model that showed good model fit (χ_(21)_^2^ = 48.323, *p* < 0.001, CFI = 0.998, RMSEA = 0.024, SRMR = 0.018). All significant path coefficients and correlations of the final RI-CLPM model are illustrated in Figure 2.

At the within-person level, we found significant autoregressive effects for EF (T1 to T2: *β* = 0.277, *p* < 0.001; T2 to T3: *β* = 0.202, *p* < 0.001), with both falling in the small-to-medium range. We also found autoregressive effects for FM, which were large in size (T1 to T2: *β* = 0.502, *p* < 0.001; T2 to T3: *β* = 0.459, *p* < 0.001). For GM, the autoregressive effect was in the small-to-medium range and only significant during the first period (T1 to T2: *β* = 0.307, *p* < 0.001).

Moving on to the cross-lagged effects, the results showed small but significant effects: FM at T1 significantly positively predicted EF at T2 (*β* = 0.077, *p* = 0.011). The same small effect occurred in the following years (T2 FM to T3 EF: *β* = 0.096, *p* = 0.012), indicating that FM skills above the participant’s mean at one-time point positively impacted EF in the following year, controlling for FM at the previous time point. In addition, FM at T1 positively predicted GM skills at T2 (*β* = 0.150, *p* < 0.001), representing a small-to-medium effect. The same effect occurred in the following years, increasing to a medium-sized effect (T2 FM to T3 GM: *β* = 0.269, *p* < 0.001).

Although the effects of fine motor (FM) skills on executive functions (EF) and FM skills on gross motor (GM) skills were statistically significant, the effect sizes were small, meeting the criteria for small effects (Cohen, 1988). Notably, the small magnitude of the within-person cross-lagged effects suggests that the longitudinal association between these constructs is primarily driven by stable trait-like between-person differences rather than short-term within-person developmental changes. We did not find cross-lagged effects between GM skills and EF.

At the between-person level, we found a significant correlation between the random intercepts of EF and GM (*r* = 0.575, *p* < 0.001). In addition, the random intercepts of EF and FM, GM and FM were also significantly correlated (*r* = 0.706, *p* < 0.001; *r* = 0.756, *p* < 0.001). 

## 4. Discussion

The present study explored the longitudinal and reciprocal relationships among EF, GM skills, and FM skills in early childhood within the Chinese cultural context. To our knowledge, this study is one of the few to investigate the dynamic interplay between EF and motor skills among preschool-aged children. Furthermore, it is pioneering in employing the Random Intercept Cross-Lagged Panel Model (RI-CLPM) to disentangle between- and within-person effects among these constructs.

Building on prior research, this longitudinal study employed three measurement points and applied RI-CLPM analyses to investigate the cross-lagged and autoregressive effects between EF and motor skills in preschool children in China. The findings largely corroborate our hypothesis that EF, GM, and FM skills are interconnected, albeit with patterns differing by age. Specifically, autoregressive effects were evident in EF and FM from T1 to T3, whereas GM exhibited autoregressive effects only between T1 and T2. Cross-lagged effects show that FM demonstrated significant cross-lagged effects on both GM skills and EF across all three assessment waves. The random intercepts of EF, GM skills, and FM skills were significantly correlated with each other.

### 4.1. The Autoregressive Effects of EF and FM

Longitudinal tracking analysis of 3- to 5-year-old children in this study revealed a significant autoregressive effect in EF development, though this effect can be characterized as a small effect, which also means that individuals’ early EF levels can effectively predict their subsequent performance. This developmental continuity is supported by a solid neurodevelopmental basis: EF development is closely associated with the maturation of the prefrontal cortex (Diamond, 2000; Tsujimoto, 2008). Significant structural and functional advancements in the prefrontal cortex during early childhood provide a key neurobiological foundation for the improvement of various EF abilities (Zelazo, 2020). Specifically, continuous myelination and the expansion and optimization of neural connections within the prefrontal cortex are core mechanisms supporting the refined development of children’s EF (Salami et al., 2003).

At the neurobiological level, the rapid development of the prefrontal cortex during the preschool period provides the foundational structural support for the stable development of EF. During this stage, gray matter volume expands substantially, synaptic pruning proceeds actively, and myelination of frontoparietal control pathways continues to increase (Huttenlocher, 1979). Together, these structural changes accelerate the maturation of prefrontal neural circuits. Building on this foundation, the neural processing pathways for core EF components (such as inhibitory control and working memory) become increasingly stable and efficient (Welsh & Pennington, 1988). As prefrontal neural circuits gradually mature, individual differences in EF shift away from transient, highly context-dependent fluctuations toward a stable, trait-like developmental pattern. This shift constitutes the neurophysiological basis from which significant autoregressive effects emerge in longitudinal studies (Fiske & Holmboe, 2019; Tamm et al., 2002).

These findings are highly consistent with existing longitudinal evidence on EF development in preschool children. C. Li et al. (2023), drawing on large-scale longitudinal data from the Chicago Head Start program, revealed a dependency of the autoregressive effect on interval length: within the preschool period, over short-interval tracking (fall to spring, approximately six months), the within-person autoregressive effect was significant and moderate in size; as the tracking interval lengthened, the accumulation of confounding factors and the heterogeneity of measurement instruments across developmental stages led to a notable attenuation of developmental continuity. Nonetheless, from the perspective of overall developmental trajectories, the preschool period remains the stage in which the autoregressive effect of EF is most pronounced. Broomell and Bell (2022), based on long-term longitudinal data spanning from infancy to late childhood, further argued that the prominence of this effect during the preschool years is essentially a product of the synergistic interplay between neurodevelopmental maturation and cumulative experiential reinforcement—an interpretation that resonates deeply with the neurodevelopmental foundations proposed in the present study.

At the environmental and experiential level, the rapid accumulation of cognitive and social experiences during the preschool period further amplifies the developmental continuity of EF, serving as a key external mechanism that continuously strengthens the autoregressive effect. As language abilities develop rapidly, daily rule-based activities increase, and cognitive challenges escalate, children’s EF is continuously consolidated and enhanced through repeated application in context (Carlson, 2005; Garon et al., 2008). This process creates a positive feedback loop of initial ability level → differential practice opportunities → gradual skill consolidation: children with higher initial EF tend to gain more opportunities for cognitive challenge and skill practice, thereby further advancing their abilities; conversely, children with weaker abilities may become caught in a cycle of relative developmental lag due to insufficient practice opportunities. For instance, in everyday kindergarten activities, children with stronger inhibitory control find it easier to follow classroom rules such as taking turns speaking and following instructions, thereby gaining greater access to structured cognitive activities (e.g., answering questions after story time, engaging in role-play games), which in turn further exercise their working memory and cognitive flexibility. It is precisely this cumulative effect of experiential reinforcement that sustains and amplifies the within-person continuity of EF development.

In terms of FM skills, the autoregressive effect was moderate to large in size: their development relies on the coordinated activation of the parietal lobe (responsible for visuomotor integration), primary motor cortex (controlling fine motor execution), and cerebellum (coordinating movements) (Goldenkoff et al., 2025; Mayhew et al., 2017). Notably, the structural and functional connections of these brain regions are not temporarily formed but show significant continuity with development. This continuity provides sustained neural support for the gradual improvement of FM skills and lays the foundation for the generation of the autoregressive effect of FM (Beck et al., 2021; X. Li et al., 2024).

During the critical period of FM development (3 to 5 years old), the development of the aforementioned brain regions is not achieved through sudden changes but through refined processing centered on the neural basis formed in the early stage. This includes enhancing myelination to improve signal transmission efficiency and optimizing synaptic refinement to polish effective connections (Giedd et al., 1999; Lebel & Beaulieu, 2011). This progressive optimization mechanism based on existing neural circuits enables the proficiency of early basic FM skills to directly affect the development quality of later neural circuits, thereby effectively predicting later FM performance, and ultimately presenting a significant autoregressive effect statistically (Dayanidhi et al., 2013; Forssberg et al., 1991).

The continuity of environmental stimulation further strengthens this autoregressive path. Sustained and diverse object manipulation experiences (e.g., handling small items) can help children establish a positive feedback loop of “skill practice → neural circuit enhancement → skill refinement” (Beck et al., 2021). Early-acquired basic FM skills, through repeated practice, not only consolidate the effectiveness of neural connections but also promote targeted improvements in the myelination process, making neural signal transmission more efficient. This bidirectional enhancement of skills and neural basis further improves the learning efficiency of complex movements in later stages, ultimately consolidating the autoregressive effect where early performance predicts later outcomes at the statistical level (Lebel & Beaulieu, 2011).

Longitudinal research on preschool-aged children provides direct empirical support for this conclusion. M. V. Wang et al. (2014), drawing on large-scale Norwegian cohort data, found that fine motor performance at age 3 significantly and positively predicted corresponding performance at age 5, with the autoregressive effect reaching a moderate size. Øksendal et al. (2022) further corroborated this stable effect and found moderate-to-high developmental stability; both findings are consistent with those of the present study. The core causes of this effect can be summarized in three points. First, FM development is strongly path-dependent. Early childhood represents a critical period during which FM skills advance rapidly from basic movements to complex skills. Early foundational abilities such as hand control and visuomotor integration are necessary prerequisites for subsequent skill acquisition, giving FM skills a strong cumulative nature (Øksendal et al., 2022). Second, everyday activities such as self-feeding, dressing, and craft play provide frequent and stable practice opportunities, creating a positive feedback loop of ability level → practice engagement → skill consolidation that continuously maintains and amplifies between-person differences (Øksendal et al., 2022; Cheng et al., 2016). Third, FM skills are overt, observable behavioral skills. Compared with more covert developmental domains such as communication and emotion, their assessment involves smaller measurement errors, and their developmental continuity can therefore be captured more stably (M. V. Wang et al., 2012).

### 4.2. The Cross-Lagged Effects of FM on EF

This study found a significant cross-lagged effect of FM skills on EF across assessments at three time points. Importantly, the magnitude of this within-person effect was small, explaining only a limited proportion of variance in EF development. However, it represents a consistent cross-year effect during the critical developmental window of 3 to 5 years. Such small cumulative effects over time may have meaningful long-term impacts on children’s cognitive and behavioral development (Adachi & Willoughby, 2015). Furthermore, stable between-person differences accounted for the majority of the variance in the FM-EF association, indicating that these constructs share common developmental antecedents. From the perspective of developmental cognitive neuroscience, this finding is consistent with the hypothesis that the core mechanism of this cross-lagged effect stems from the sustained shaping and gradual optimization of EF-related neural networks by FM skills, with this shaping exhibiting a cumulative cross-time effect. According to Diamond’s (2000) theory, the proficient execution of FM relies on the coordinated activation of the cerebellum and prefrontal cortex. This sensorimotor learning process is not a one-time neural regulation but continuously strengthens the functional connectivity and information transmission efficiency of the prefrontal-cerebellar network through repeated motor practice. As the core neural basis of EF, the development and maturation of this network exhibit significant continuity and path dependence (Ridler et al., 2006): the proficient mastery of early FM skills initiates the optimization process of this network, and the improvement of network function provides necessary support for subsequent EF development. This model of “motor practice → network optimization → cognitive enhancement” provides a plausible theoretical explanation for the core logic of FM predicting EF across three waves. FM performance in the previous wave may reflect the current level of neural network optimization, which could become the foundation for EF development in the next wave, ultimately forming a stable cross-time predictive relationship. From the perspective of everyday behavioral mechanisms, FM activities in early childhood naturally place demands on EF. Take puzzles as an example: children must continuously inhibit the impulse to pick up incorrect pieces (inhibitory control), remember previously attempted combinations while trying new pieces (working memory), and flexibly switch strategies when stuck (cognitive flexibility). Similarly, common kindergarten activities such as bead threading and paper cutting require children to simultaneously deploy EF resources such as attentional control and error correction while executing FM actions. Although the facilitating effect of a single activity on EF may be small, daily repeated operational experiences during the critical period of rapid FM development at ages 3–5 can produce cumulative shaping effects. This represents the potential behavioral explanation for why the cross-lagged effect in this study, although small in size, remained stable across waves.

### 4.3. The Cross-Lagged Effects of FM on GM

Notably, this study found that FM has a significant positive predictive effect on later GM skills, and this conclusion can be indirectly supported by the previous research (Souza et al., 2010). Their longitudinal tracking of infants aged 12–17 months showed that FM development was significantly superior to GM development in the same developmental stage, and this advantage became more prominent in subsequent assessments. This prioritization in development of FM has laid an important foundation for its longitudinal positive prediction of GM: the relatively mature development of early FM can provide necessary ability preparation for the development of GM in this and subsequent stages, thus resulting in an observable positive predictive effect.

The results of this study are inconsistent with the conclusion by Piek et al. (2008) that the developmental trajectory of FM has no significant prediction of later motor skills. Piek et al. used the FM and GM subscales of the ASQ (Ages and Stages Questionnaire), in which parents report whether basic motor milestones have been achieved (e.g., grasping objects, rolling over, crawling). These subscales are relatively coarse, focusing on the presence or absence of motor abilities rather than their sophistication. In contrast, the measure used in the present study places greater emphasis on indicators related to skill transfer, such as force control during grasping and visuomotor precision. It is therefore more sensitive to detecting a predictive effect of FM skills on GM skills. Moreover, Piek et al. tracked infants aged 0–4 years and predicted GM performance at 6–11 years, a period during which FM skills are still nascent and GM assessments primarily target basic mobility. In contrast, the present study focused on ages 3–5 years—a window in which FM skills undergo rapid differentiation and cross-domain transfer effects are most active.

The cross-cultural context further strengthens the predictive power of FM on GM in this study. Research has shown that compared with children from Western countries such as the United States and the United Kingdom, Chinese children often exhibit significant advantages in FM skills (especially in grasping and manipulation, visuomotor integration dimensions) (Chow et al., 2001; Chui et al., 2007; Martzog et al., 2019). Chow et al. (2001), using the Movement Assessment Battery for Children (Movement ABC), found that Hong Kong children aged 4–6 significantly outperformed their American peers on manual dexterity tasks such as coin dropping and trail tracing. Chui et al. (2007), using the Bruininks-Oseretsky Test of Motor Proficiency (BOTMP), further demonstrated that Hong Kong children aged 6–10 scored significantly higher than American norms on dimensions of visual-motor control, upper-limb speed, and dexterity. This FM advantage is rooted in early child-rearing and educational practices in East Asian cultures: Chinese children typically begin learning to use chopsticks around age 2 and start practicing Chinese character writing around age 3. Both chopstick use and character writing impose high-frequency, high-demand training on finger force control, hand–eye coordination, and visuospatial matching. Such daily FM practice not only elevates the overall level of FM skills, but also repeatedly exercises foundational general abilities such as spatial perception, sensorimotor feedback regulation, and motor sequence planning (D. Wu & Sun, 2020). These abilities, in turn, are precisely the core supports for the development of complex and refined GM skills (van der Fels et al., 2020). Therefore, a stronger FM foundation and greater transferable capacity reserves make the longitudinal predictive effect of FM skills on GM skills more readily detectable, and more prominent in magnitude among Chinese children.

### 4.4. The Cross-Lagged Effects of EF to Motor Skills

In contrast to the significant cross-lagged effects of FM on EF, the present study found no significant cross-lagged effects of EF on subsequent FM or GM skills, which aligns more closely with the unidirectional prediction that motor skills serve as the foundation for cognitive development, as proposed by Automation Theory and Piaget’s Cognitive Development Theory (Cameron et al., 2015; Piaget, 1952). This result may be explained by three factors: First, this study used parent reports to assess both EF and motor skills. A limitation of this approach is that parent reports and performance-based measures reflect different levels of the construct (Toplak et al., 2013) and are often somewhat disconnected from laboratory task-based assessments (Ten Eycke & Dewey, 2016). This discrepancy is even more pronounced in clinical populations (Sankalaite et al., 2025), suggesting that parent reports and behavioral tasks may capture different facets of EF. Second, the 3–5 year period is a phase of rapid motor skill development, during which motor acquisition depends more on sensorimotor experience accumulation than on higher-order cognitive control (Masters et al., 2013). Masters et al. (2013) argued that preschool children can acquire complex motor skills through error-reduced practice environments without relying on explicit cognitive resources such as working memory. Using a large Norwegian longitudinal cohort, Øksendal et al. (2022) found that motor skills in 3- to 5-year-old children exhibit substantial longitudinal stability. These robust autoregressive effects indicate that early motor competence is a strong predictor of later motor performance. This pattern suggests that early motor development is primarily sustained by the cumulative accretion of sensorimotor experience, rather than by transient cognitive control resources, thereby further supporting the above argument. Third, the association between the two constructs is primarily driven by between-person differences arising from common antecedents such as family environment (Obradović et al., 2016; Williams et al., 2026). When this between-person variance component is statistically partitioned by the RI-CLPM model, the remaining within-person dynamic effects are relatively weak and fail to reach statistical significance.

### 4.5. The Relationship Between GM Skill and Executive Function

Furthermore, our examination of the bidirectional relationship between GM skills and EF in children aged 3 to 5 years revealed no statistically significant association. First, at the neural level, FM tasks (e.g., manual manipulation) activate the prefrontal–cerebellar circuit more directly than GM tasks (e.g., running and jumping) (Stoodley, 2011). Thus, FM skills shape EF more directly; in contrast, GM skills rely more heavily on subcortical motor circuits with a higher degree of automaticity, placing relatively limited demands on higher-order cognitive control. The cross-sectional study by Maurer and Roebers (2019) provided empirical support for this view: FM skills were significantly associated with EF regardless of task difficulty, whereas GM skills were only associated with EF at higher levels of task difficulty. This implies that GM activities at routine intensity levels in daily contexts may not be sufficient to produce a detectable shaping effect on EF.

Second, from a developmental stage perspective, GM skills in children aged 3–5 are still in the phase of establishing basic movement patterns (Iivonen et al., 2011; Ruiz-Esteban et al., 2020) and have not yet entered a stage requiring highly cognitively demanding complex movement strategies (e.g., tactical decision-making in team ball games). Consequently, GM development at this age depends more on physical maturation and the accumulation of sensorimotor experience than on active regulation by EF (Thelen & Smith, 1994). This interpretation aligns with the present study’s finding of no significant predictive path from EF to GM skills—the top-down influence of EF on motor skills may only emerge gradually at school age, when motor and cognitive demands become deeply coupled.

Third, the association between GM skills and EF may operate largely through indirect mediating pathways rather than through direct bidirectional prediction. For instance, Cameron et al. (2016) noted that GM skills are closely related to children’s social competence and physical health, whereas FM skills show stronger links with academic achievement. Son and Meisels (2006), analyzing data from the Early Childhood Longitudinal Study, also found that although both FM and GM skills at kindergarten entry predicted Grade 1 reading and mathematics achievement, the effect sizes for GM skills were considerably smaller. Taken together, a more plausible inference is that the contribution of GM skills to cognitive development is transmitted through indirect pathways such as enhanced social participation and improved physical health, rather than directly affecting the neurocognitive processes underlying EF.

### 4.6. Limitations and Future Directions

This study has several limitations. First, in terms of measurement methods, it mainly relies on parent reports. Although this method can effectively reflect children’s behavioral performance in daily environments and has good ecological validity, it may have differences with the results of standardized behavioral assessments or laboratory neuropsychological tests (Toplak et al., 2013). Second, regarding the generalizability of the conclusions, this study is based on large-sample data from a specific cultural context. Since children’s early developmental trajectories are significantly influenced by cultural customs and educational practices, whether the predictive patterns of FM skills on EF and GM skills revealed in this study apply to other cultural contexts remains to be verified through cross-cultural comparative studies. Additionally, we only assessed children once per year, which was too infrequent to capture the small, rapid changes that occur in early development.

Addressing the above limitations, future research can be further advanced in the following directions: First, combine multi-informant assessments with neuroimaging techniques: integrate teacher reports and direct behavioral measures to more comprehensively assess EF and motor skills, and use fNIRS and MRI to directly examine the neural mechanisms underlying the “fine motor-cerebellum-prefrontal cortex” pathway in cognitive-motor development. Second, conduct cross-cultural comparative studies to examine whether the observed relationships between FM skills, GM skills, and EF generalize to other cultural contexts. Third, conduct longer-term longitudinal studies with more frequent assessments (e.g., every 6 months) to better capture the dynamic relationships between motor skills and EF in early childhood, and examine how these preschool associations predict later academic achievement and socio-emotional development.

## 5. Conclusions

In conclusion, this study offers a comprehensive analysis of the longitudinal relationships between EF and motor skills during early childhood in Chinese culture. Through the application of RI-CLPM, we elucidated the intricate dynamics between these constructs, revealed the reinforcing role of shared cultural socialization practices on between-person associations, and provided key empirical evidence for the cross-cultural universality of the theory of motor–cognition co-development. In terms of theoretical contributions, this study not only extends the boundary of cultural applicability of the bidirectional relationship between EF and motor skills, but also, through its methodologically rigorous design, disentangles individual differences and developmental dynamics that may have been confounded in prior research, thereby responding to the field’s call to distinguish between-person from within-person effects. These findings carry clear implications for early education: educational institutions should organically incorporate activities rich in fine motor components—such as chopstick use, craftwork, and drawing—into daily curricula to promote EF development. Policymakers may consider integrating motor skill development into preschool education quality assessment systems, thereby advancing the implementation of the principle of coordinated physical and mental development. The results are also highly consistent with current international trends in early childhood education that emphasize whole-child education and learning through play, providing evidence-based support for the design of developmentally appropriate early childhood programs.

## Figures and Tables

**Table 1:** Descriptive Statistics and Correlations among variables.

	M	SD	1	2	3	4	5	6	7	8	9	10	11	12
Time 1														
1. gender	1.49	0.50	-											
2. FEDU	8.91	206.84	0.02	-										
3. MEDU	8.89	206.84	0.02	1.00 **	-									
4. EF	2.85	0.58	0.09 **	0.02	0.02	-								
5. gross	2.85	0.44	−0.02	0.01	0.01	0.43 **	-							
6. fine	2.30	0.45	0.23 **	0.02	0.02	0.48 **	0.65 **	-						
Time 2														
7. EF	3.16	0.56	0.10 **	0.02	0.02	0.60 **	0.35 **	0.39 **	-					
8. gross	3.29	0.38	−0.01	0.03	0.02	0.39 **	0.68 **	0.47 **	0.46 **	-				
9. fine	2.94	0.45	0.23 **	0.02	0.02	0.42 **	0.50 **	0.67 **	0.50 **	0.63 **	-			
Time 3														
10. EF	3.40	0.49	0.10 **	0.01	0.01	0.52 **	0.30 **	0.33 **	0.61 **	0.38 **	0.40 **	-		
11. gross	3.57	0.31	0.00	0.01	0.01	0.37 **	0.60 **	0.40 **	0.38 **	0.71 **	0.51 **	0.48 **	-	
12. fine	3.45	0.35	0.18 **	0.01	0.01	0.41 **	0.47 **	0.55 **	0.44 **	0.54 **	0.70 **	0.52 **	0.69 **	-

Note. EF = executive function; Gross = Gross motor skill; Fine = Fine motor skill; ** *p* < 0.01.

**Table 2:** Model comparison and fit for the RI-CLPM without covariates.

Model	χ^2^	df	RMSEA	CFI	TLI	SRMR	Δχ^2^	Δdf	Comparison Model	*p*
M1: Baseline model (unconstrained model)	28.781	3	0.061	0.998	0.976	0.018				
M2: Model with autoregressive paths fixed to be time-invariant	139.411	6	0.098	0.99	0.938	0.065	110.63	3	M2 vs. M1	<0.001
**M3: Model with cross-lagged paths fixed to be time-invariant**	**45.157**	**9**	**0.041**	**0.997**	**0.989**	**0.021**	**16.376**	**6**	**M3 vs** **. M1**	**0.0119**
M4: Model with autoregressive and cross-lagged paths fixed to be time-invariant	159.982	12	0.073	0.988	0.965	0.075	114.825	3	M4 vs. M3	<0.001

Note: Bold indicates final selected model; RMSEA < 0.05 indicates good fit, <0.08 indicates acceptable fit; CFI and TLI > 0.95 indicate good fit; SRMR < 0.08 indicates good fit.

## Data Availability

The data analyzed in this study are publicly available from the Taiwan Survey Research Data Archive, Center for Survey Research, Research Center for Humanities and Social Sciences, Academia Sinica (doi: 10.6141/TW-SRDA-D00168-2).

## Abbreviations

The following abbreviations are used in this manuscript:

ADHD	Attention-Deficit/Hyperactivity Disorder
DCD	Developmental Coordination Disorder
KIT	Kids in Taiwan: National Longitudinal Study of Child Development and Care
EF	Executive Function
GM	Gross Motor
FM	Fine Motor
EM	Expectation-Maximization
DMSSPC	Developmental Motor Screening Scale for Preschool Children
fNIRS	functional Near-Infrared Spectroscopy
CFI	Comparative Fit Index
MCAR	Missing Completely At Random
RI-CLPM	Random Intercepts Cross-Lagged Panel Model
RMSEA	Root Mean Square Error of Approximation
SRMR	Standardized Root Mean Square Residual
TLI	Tucker-Lewis Index
T1	Time Point 1
T2	Time Point 2
T3	Time Point 3
